# COP1 Jointly Modulates Cytoskeletal Processes and Electrophysiological Responses Required for Stomatal Closure

**DOI:** 10.1093/mp/ssu065

**Published:** 2014-05-23

**Authors:** Rajnish Khanna, Junlin Li, Tong-Seung Tseng, Julian I. Schroeder, David W. Ehrhardt, Winslow R. Briggs

**Affiliations:** ^a^Department of Plant Biology, Carnegie Institution for Science, 260 Panama Street, Stanford, CA 94305, USA; ^b^Division of Biological Sciences, University of California San Diego, 9500 Gilman Drive, La Jolla, CA 92093-0116, USA; ^c^ Present address: College of Forest Resources and Environment, Nanjing Forestry University, Nanjing, 210037, China

**Keywords:** stomatal function, microtubule dynamics, hormone signaling.

## Abstract

Reorganization of the cortical microtubule cytoskeleton is critical for guard cell function. Here, we investigate how environmental and hormonal signals cause these rearrangements and find that COP1, a RING-finger-type ubiquitin E3 ligase, is required for degradation of tubulin, likely by the 26S proteasome. This degradation is required for stomatal closing. In addition to regulating the cytoskeleton, we show that *cop1* mutation impaired the activity of S-type anion channels, which are critical for stomatal closure. Thus, COP1 is revealed as a potential coordinator of cytoskeletal and electrophysiological activities required for guard cell function.

## SUMMARY

COP1-mediated proteolysis is required for stomatal closure. In guard cells, COP1 function is linked to microtubule destabilization and the activity of S-type anion channels leading to stomatal closure.

## INTRODUCTION

Higher plants have developed finely tuned mechanisms to regulate gas exchange through pores known as stomata in the leaves and stems. Pairs of specialized cells known as guard cells form stomatal pores. Embedded in the epidermis of photosynthetic organs, stomatal pores control the influx of atmospheric carbon dioxide (CO_2_) required for photosynthesis, release of oxygen, and the outflow of water vapor through transpiration (Kim et al., 2010; Kollist et al., 2014). Stomatal function regulates gas exchange, drives water and nutrients from the roots to the leaves and growing shoots, and prevents excessive water loss. Stomatal activity is thought to have played a central role in transforming the Earth System, and it may influence future climate and weather, as revealed by global modeling of interactions between land and atmosphere (Berry et al., 2010).

The specialized guard cells have developed intrinsic mechanisms to swell or shrink, promoting open or closed stomatal pores in response to environmental cues such as changes in light conditions, atmospheric CO_2_, temperature, humidity, soil water availability, and endogenous plant hormonal stimuli. Previous studies have shown that stomatal movements require coordinated molecular and physiological events in guard cells, including changes in ion exchange (Kim et al., 2010), turgor, altered gene expression (Gray, 2005), protein modification, and rearrangement of actin filaments and microtubules (Hetherington and Woodward, 2003; Eisinger et al., 2012a, 2012b). Slight effects of actin antagonists on stomatal movement (Hwang et al., 2000; Hwang and Lee, 2001; MacRobbie and Kurup, 2007) and reorganization of actin filaments in response to light and ABA (Eun and Lee, 1997) have been shown.

Light is an essential regulator of stomatal opening. Stomata are closed in the dark. It has been shown that phototropins (PHOT1 and PHOT2) proteins mediate blue light-induced stomatal opening (Kinoshita et al., 2001; Mao et al., 2005). Light signaling activates H^+^-ATPases leading to hyperpolarization of the plasma membrane, which drives uptake of K^+^ through voltage-dependent inward-rectifying K^+^ channels. Increase in guard cell osmotic potential leads to accumulation of water, cell swelling, and stomatal pore opening (Kim et al., 2010).

Stomatal closure is achieved by reduction in guard cell turgor. Under conditions of water stress, abscisic acid (ABA) induces an increase in cytosolic calcium ([Ca^2+^]_cyt_) together with Ca^2+^-independent mechanisms, resulting in the activation of anion channels (Pei et al., 1997; Mustilli et al., 2002; Barbier-Brygoo et al., 2011), including the slow (S-type) channel SLAC1 (SLOW ANION CHANNEL 1) (Vahisalu et al., 2008; Kollist et al., 2011), which causes depolarization of the plasma membrane (Schroeder et al., 1987), suppression of K^+^_in_ channels and increased K^+^_out_ channel activity (Schroeder et al., 1987; Blatt and Armstrong, 1993; Miedema and Assmann, 1996; Hosy et al., 2003), leading to a net K^+^ efflux from guard cells, loss of turgor, and stomatal closure (Schroeder and Hagiwara, 1989). Stomatal movements are mediated through a tight control of a network of ion channels in the plasma membrane of guard cells, and anion channels (Keller et al., 1989; Schroeder and Hagiwara, 1989; Pei et al., 1997) and the SLAC1 protein play critical roles in stomatal closure (as well as in stomatal opening) by directly or indirectly altering ion movement (Negi et al., 2008; Vahisalu et al., 2008). The *slac1* mutants are impaired in stomatal closure in response to several factors, including CO_2_, calcium, ABA, and light-to-dark transitions (Negi et al., 2008; Vahisalu et al., 2008). Recently, Laanemets et al. (2013) reported that *slac1* mutant alleles had reduced activities of K^+^_in_ channels, which could be reversed by lowering [Ca^2+^]_cyt_, indicating that K^+^_in_ channel sensitivity to [Ca^2+^]_cyt_ was elevated in *slac1* mutants, consistently with their reduced stomatal opening response to low CO_2_ and light.

While great progress has been made in understanding the molecular mechanisms underlying guard cell ion channel functions and regulation, much remains to be learned about the cellular processes that lie between signal perception and the activities of channels and pumps, and how they orchestrate stomatal movements. A number of studies, including data we present here, have indicated important roles for transcription, protein turnover, and the cytoskeleton.

Mao et al. (2005) showed that CONSTITUTIVELY PHOTOMORPHOGENIC 1 (COP1), a RING-finger-type E3 ubiquitin ligase, functions downstream of cryptochrome and phototropin photoreceptors to repress stomatal opening. COP1 is a known suppressor of photomorphogenesis and photoperiodic flowering, acting through ubiquitylation and targeted degradation of several light-signaling factors including HY5 (LONG HYPOCOTYL 5) (Osterlund et al., 2000; Saijo et al., 2003), HYH (HY5-HOMOLOG) (Holm et al., 2002), LAF1 (LONG AFTER FAR-RED LIGHT1) (Seo et al., 2003), HFR1 (LONG HYPOCOTYL IN FAR-RED1) (Jang et al., 2005), and CO (CONSTANS) (Liu et al., 2008). However, it is not known how COP1 functions to suppress stomatal opening in the dark.

Cortical microtubule arrays are dynamic structures that are essential for regulating cellular morphogenesis, acting in part to position the secretion of cellulose synthase (Gutierrez et al., 2009) and to guide the trajectories of these transmembrane protein complexes as they deposit cellulose into the cell wall. As mature guard cell function is intimately related to their shape and to the expansion and shrinking of their cell walls, cytoskeletal direction of cell wall assembly and shape should be important for building guard cells with appropriate functional properties. Changes in cytoskeletal organization have also been observed and correlated with stomatal movements (Eisinger et al., 2012a, 2012b). Consistently, evidence was observed for increased assembly of cortical arrays as stomata opened, and destabilization as they closed. Importantly, when the stability of cortical arrays was manipulated by pharmacological agents, artificial disassembly prevented stomatal opening, and artificial stabilization prevented closure, indicating that the organization and function of these arrays play an essential and previously unknown role in guard cell function (Eisinger et al., 2012a, 2012b). Cytoskeletal organization is also known to be sensitive to some of the same signals that affect guard cell function, such as blue light, which has recently been shown to drive reorientation of cortical arrays in etiolated *Arabidopsis* hypocotyls by a mechanism dependent on phototropin-stimulated microtubule severing by the protein katanin (Lindeboom et al., 2013). The mechanisms by which cytoskeletal organization is regulated by signals that control stomatal movements and the role or roles that the cytoskeleton plays in guard cell function remain to be determined.

In this study, we combined genetic analysis, quantitative live cell imaging and electrophysiological studies to show that COP1 function is required for microtubule protein degradation and normal anion channel activation during stomtatal closure. These results provide links between biochemical mechanisms regulating electrophysiological and cytoskeletal changes in guard cells.

## RESULTS

### Inhibition of Proteolysis Impairs ABA-Induced Stomatal Closure

Guard cells undergo physiological and structural transformations during stomatal closure over a period of 30–40min. These changes may require synthesis of factors regulating stomatal closure and destabilization of components required for stomatal opening, such as microtubule arrays. The decline in microtubule bundles observed during stomatal closure might be caused by a decrease in assembly, an increase in disassembly, or both. Observations of plus end number and growth rates indicated that assembly was not measurably affected, so we hypothesized that disassembly, likely from free minus ends, was primarily responsible for changes in array structure (Eisinger et al., 2012a, 2012b). The observation that total GFP–TUA6 fluorescence fell at a faster rate during stomatal closure than could be accounted for by photobleaching suggested that tubulin itself might be degraded during closure—a process that could contribute to the loss of bundles.

**Figure 1:**
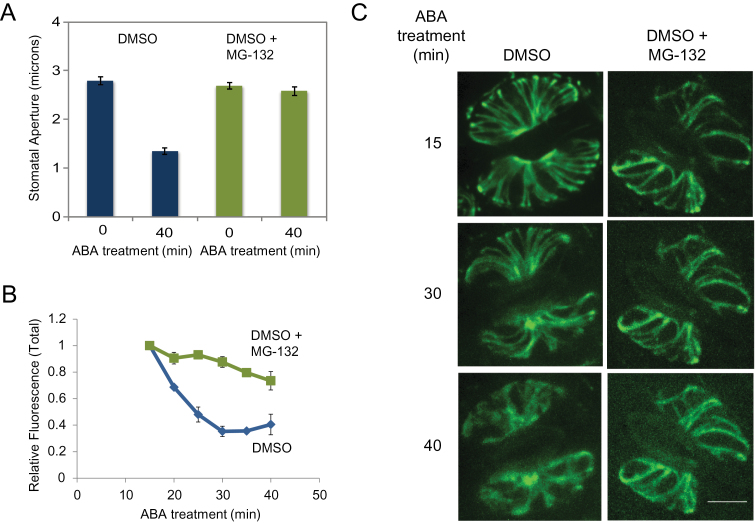
Inhibition of Proteolysis Impairs ABA-Induced Stomatal Closure and Decreases in GFP-Tubulin Fluorescence. **(A)** Stomatal apertures (*n* > 50) are plotted from leaf peels either pre-treated with DMSO or (DMSO + 50 μM MG-132) for 1 h, followed by 10 μM ABA treatment as indicated. **(B)** Relative changes in total GFP-tubulin fluorescence in guard cells is plotted over time during the ABA treatment. Inhibition of proteolysis attenuated the loss of total fluorescence in response to ABA treatment. **(C)** Confocal images of GFP:tubulin-labeled guard cells either pre-treated with DMSO, or DMSO + MG-132. Data shown are from one of three independent repeats, each with similar results. Images were taken as described in the ‘Methods’ section. Error bars show ±SE. Scale bar = 5 μm.

**Figure 2:**
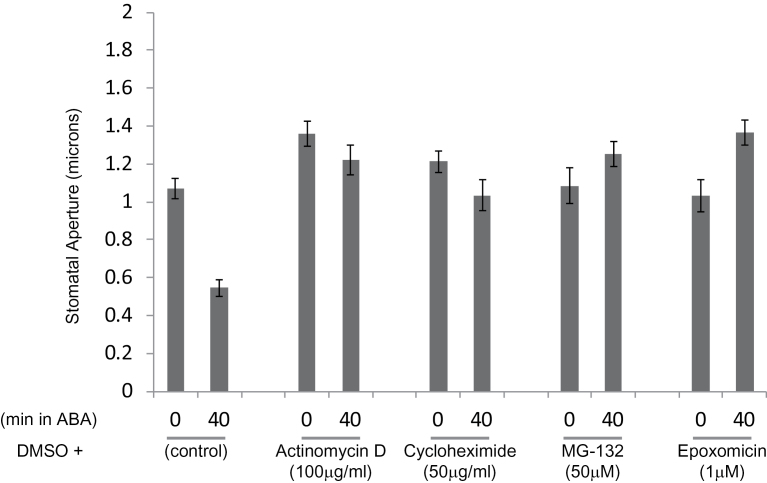
ABA-Induced Stomatal Closure Requires *De Novo* Transcription and Translation, and Proteolysis. Leaf peels from *GFP–TUA6* control lines were pre-treated (1 h) either with DMSO alone (control), or with various inhibitors including Actinomycin D (transcriptional), Cycloheximide (translational), MG-132, or epoxomycin (proteolysis inhibitors; reversible or irreversible, respectively). Following pre-treatment with inhibitor, leaf peels were exposed to 10 mM ABA for 40min. Stomatal apertures are plotted before (0) and after ABA (40) treatment. Data shown are from one of two independent experiments each with (*n* > 50). Error bars show ±SE.

To test whether protein degradation may be required for stomatal closure and for the observed changes in cytoskeletal structure, we pre-treated epidermal peels with either the 26S proteasome inhibitor MG-132 or a control solution, followed by ABA treatment to induce stomatal closure. Pre-treatment with MG-132 was sufficient to block ABA-induced stomatal closure (Figure 1A), suggesting that 26S proteasome activity plays a role in this process. In addition to blocking stomatal closure, pre-treatment with MG-132 significantly reduced the ABA-induced decline of total GFP-tubulin fluorescence (Figure 1B) and prevented loss of radial microtubule bundles (Figure 1C). These results indicate that proteolysis by the 26S proteasome is also required for the destabilization of the cortical array and loss of tagged tubulin during closure. As a further test, we pre-treated cells with epoxomycin, a potent irreversible proteasome inhibitor. As with MG-132, pre-treatment with epoxomycin blocked stomatal closure in response to ABA (Figure 2). Together with the previous manipulations of microtubule assembly using stabilizing and destabilizing drugs (Eisinger et al., 2012a), these results indicate that microtubule array destabilization and loss of tubulin protein via the 26S proteasome pathway play a critical role in stomatal function during closing.

To determine whether *de novo* transcription and translation were also required for stomatal closure in response to ABA, we pre-treated guard cells in leaf peels with Actinimycin D or Cycloheximide. Both treatments inhibited stomatal response to ABA treatment (Figure 2), indicating a role for *de novo* transcription and translation during stomatal closure. ABA is known to mediate transcriptional and posttranscriptional responses and our results suggest that ABA may trigger transcription and translation of downstream components involved in stomatal closure. These findings are consistent with previously reported ABA-mediated transcriptional regulation of signaling components in guard cells (Leonhardt et al., 2004; Wang et al., 2011a), and are supported by the similarities between responses to ABA and quinabactin, a sulfonamide ABA agonist, in causing stomatal closure and regulating gene expression in guard cells (Okamoto et al., 2013).

### Loss of COP1 Activity Impairs ABA-Induced Stomatal Closure

To investigate how the cytoskeleton is destabilized by signals stimulating stomatal closure, we considered a possible role for COP1. COP1 is an E3 ubiquitin protein ligase required for targeted degradation of photomorphogenesis-promoting factors (Wei and Deng, 1996; Hardtke and Deng, 2000), and is also required for stomatal closure in darkness (Mao et al., 2005). To determine whether COP1 may have a role in guard cell microtubule destabilization, we crossed the GFP–TUA6 line with the *cop1-4* mutant and isolated homozygous GFP–TUA6,*cop1-4* lines (see the ‘Methods’ section). While COP1 activity was known to be required for closure in the dark, it was not known whether COP1 activity is required in general for stomatal closure, or whether this requirement is specific for light-related signaling.

**Figure 3:**
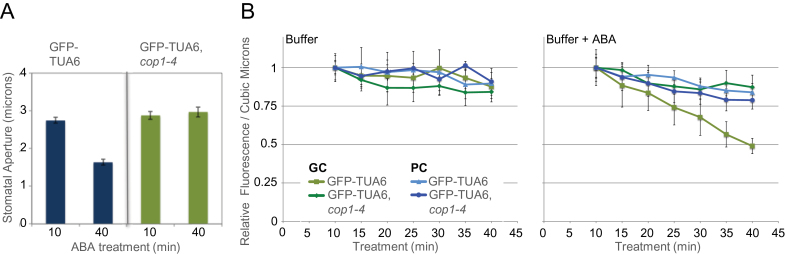
Loss of COP1 Activity Impairs ABA-Induced Stomatal Closure and Decreases in GFP-Tubulin Fluorescence. **(A)** Stomatal apertures (*n* > 50) are plotted from leaf peels from GFP–TUA6 and GFP–TUA6, *cop1* mutant plants treated with 10 mM ABA as indicated. **(B)** Relative changes in total GFP-tubulin fluorescence in guard cells (GC) and pavement cells (PC) is plotted over time during the ABA treatment. Data shown are from three independent experiments. Error bars show ±SE.

**Figure 4:**
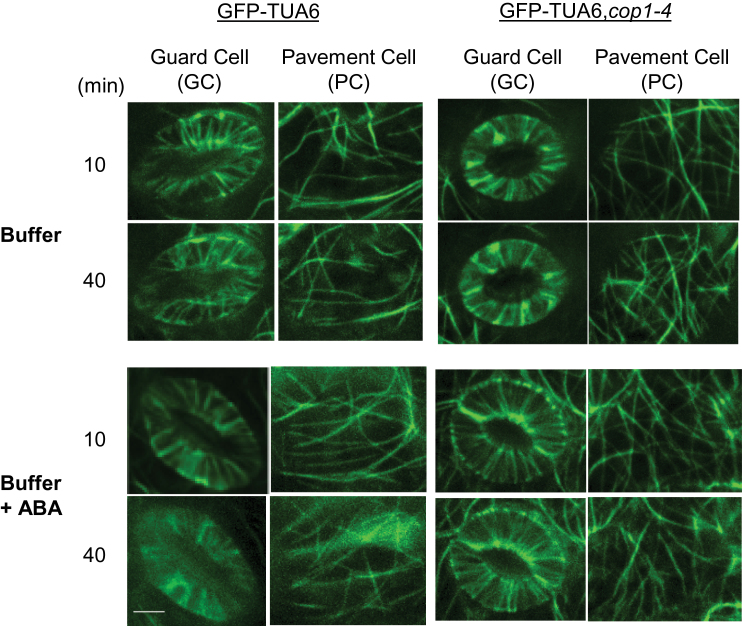
COP1 Activity Is Required for ABA-Induced Loss of Labeled Tubulin and Radial Array Structure. Confocal images of GFP:tubulin-labeled guard cells in WT and *cop1* mutant backgrounds. Data shown are from one of the three independent experiments with similar results. Images were taken as described in the ‘Methods’ section. Scale bar = 5 μm.

Stomata in GFP–TUA6,*cop1-4* did not close in response to a 40-min treatment with 10 μM ABA (Figure 3A), indicating that COP1 function is also required for ABA-induced stomatal closure. Likewise, total fluorescence in GFP–TUA6,*cop1-4* guard cells failed to fall as rapidly following ABA treatment as it did in WT cells expressing GFP–TUA6. In fact, changes in the fluorescence of GFP–TUA6,*cop1-4* guard cells were not distinguishable from those in either pavement cells of the same plants, or in plants not treated with ABA, suggesting that the relatively slow loss of signal in these cells was likely caused by photobleaching (Figure 3B). Further, microtubule structure and distribution remained relatively unaltered in GFP–TUA6,*cop1-4* guard cells, pavement cells, and cells not treated with ABA (Figure 4). Thus, both ABA treatment and COP1 function were required to cause loss of labeled tubulin and radial array structure. These data indicate that COP1 activity is required for microtubule destabilization during ABA-induced stomatal closure.

### Disruption of Microtubules in *cop1-4* Mutant Guard Cells Leads to Stomatal Closure

**Figure 5:**
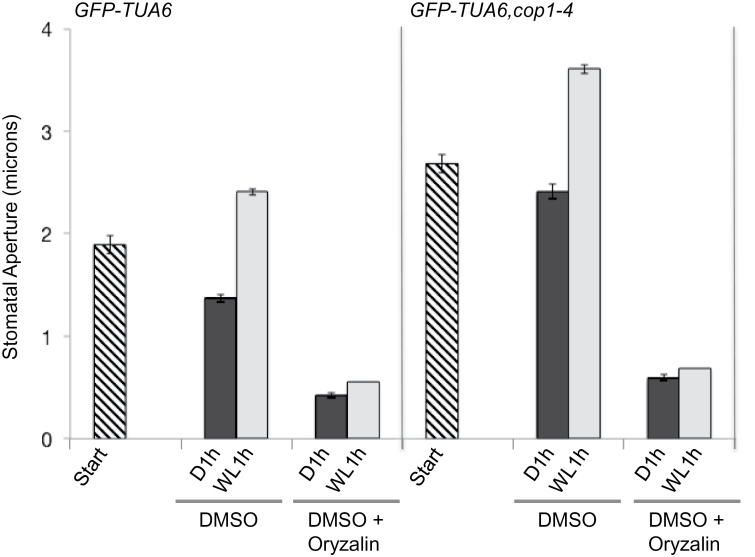
Disruption of Microtubules with Oryzalin Treatment Restores Stomatal Closure in *cop1-4* Mutants. Stomatal apertures were measured on leaf peels prior to treatment with oryzalin (Start), and after incubating either in DMSO or DMSO + 0.1 mM oryzalin in dark (D1h) and then white light (WL1h). Data shown are from one of three independent experiments each with (*n* > 40). Error bars show ±SE.

We tested whether disruption of microtubules by oryzalin in the *cop1-4* mutant background was sufficient to restore stomatal closure. Leaf epidermal peels from GFP–TUA6 and GFP–TUA6,*cop1-4* plants were treated either with DMSO (control) or with DMSO + oryzalin in buffer containing 0.1 M KCl. It was previously reported that 0.1 M KCl promoted stomatal opening (Eisinger et al., 2012b). Therefore, the buffer conditions were favorable for open stomata. Stomatal apertures were measured on peels at the start of the experiment before oryzalin treatment, after 1 h of darkness, and a third time following 1 h of white light (100 μmol m^–2^ s^–1^). Stomata in GFP–TUA6 leaf peels not treated with oryzalin closed slightly in the dark and opened more widely than the starting width in response to the white light treatment, whereas stomata in GFP–TUA6 peels treated with oryzalin were closed regardless of the light treatment (Figure 5). Consistently with previous reports, stomata in GFP–TUA6,*cop1-4* leaf peels were open in the dark, and were more open than those in WT tissue at both the start and after exposure to white light (Figure 5). Disruption of microtubules with oryzalin caused stomatal closure in the *cop1-4* background similar to the response in control stomata (Figure 5). These results indicate that COP1 activity and destabilization of microtubules play critical roles in stomatal closure. It is not yet known whether COP1 acts directly on tubulin or more indirectly by controlling one or more assembly factors, but COP1-dependent destabilization of microtubules is likely to be involved in both dark and ABA-induced stomatal closure.

### COP1 Modulates Stomatal Shape and Influences the Range of Stomatal Movement

**Figure 6:**
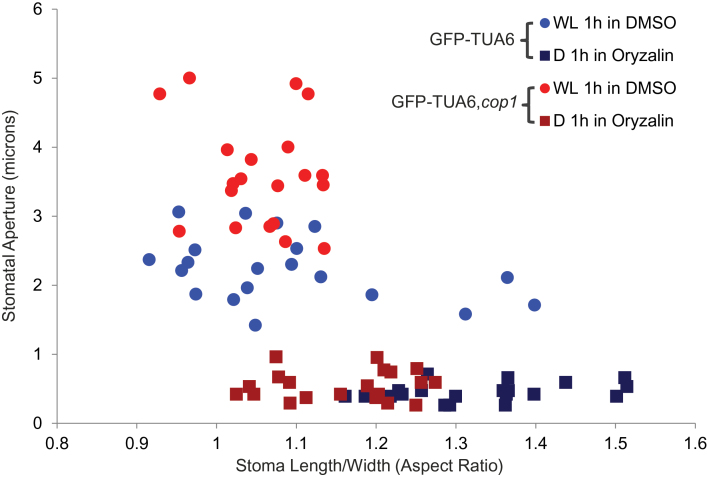
*cop1-4* Stomata Have Relatively Larger Stomatal Apertures and Maintain the Shape of Open Stomata. Aspect ratios (L/W) of individual stomata from Figure 5 are plotted with their stomatal apertures (microns) in either closed (squares, D1h in DMSO + oryzalin), or in open (circles, WL1h in DMSO) positions. The *GFP–TUA6*,*cop1-4* stomata display relatively larger stomatal apertures (open positions), and smaller aspect ratios (closed positions) in comparison to GFP–TUA6 stomata (*n* = 20).

Guard cell shape is dynamic and changes between closed and open stomata. Supplemental Figure 1A shows representative images of GFP–TUA6 and GFP–TUA6,*cop1-4* stomata in closed and open positions. Aspect ration (length/width) was calculated using length and width measurements of open and closed stomata (Supplemental Figure 1B), as described. Normally, *Arabidopsis* stomata are more elongated when closed with a higher length/width (L/W) aspect ratio than when they are open (Supplemental Figures 1 and 2). Open stomata have a more rounded shape, with the aspect ratio near 1.0 (Supplemental Figure 2B). Since the GFP–TUA6,*cop1-4* stomata lacked responsiveness to ABA treatment (Figure 3A), we used oryzalin to induce stomatal closure in GFP–TUA6,*cop1-4* and in control stomata. The changes in the aspect ratios between closed and open positions of GFP–TUA6 stomata (18.18%, *p* < 0.001) and GFP–TUA6,*cop1-4* stomata (8.62%, *p* < 0.001) showed a marked difference in the dynamic range of stomatal movement in the two genotypes (Supplemental Figure 2B). These data indicate that stomata in the *cop1* background were significantly different in shape during closure. Direct comparison of stomatal apertures and aspect ratios of stomata revealed that GFP–TUA6 stomata were more elongated when they were closed (Figure 6). GFP–TUA6,*cop1-4* stomata had relatively larger stomatal apertures and they maintained a more rounded shape (aspect ratio closer to 1) even when they were closed (Figure 6). These data suggest that COP1 modulates stomatal shape and a loss of its function results in reduction of the dynamic range of stomatal movements, locking the stomata into more open shapes.

### The *cop1* Mutation Impairs Ca^2+^ Activation of S-Type Anion Channel Currents, but Exhibits No Significant Change in Activation of Inward K^+^ Channel Currents in *cop1* Guard Cell Protoplasts

**Figure 7:**
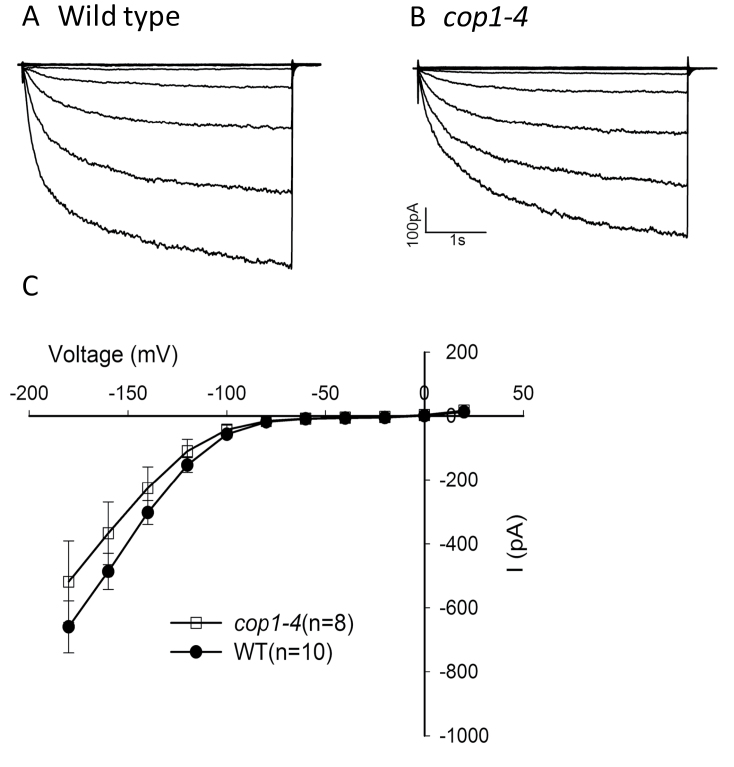
*cop1-4* Mutants Do Not Significantly Affect Inward-Rectifying K^+^ Channel Activities in *Arabidopsis* Guard Cells. Whole-cell recordings of inward K^+^ currents with the free cytosolic [Ca^2+^] buffered to 100 nM in the presence of 30 mM KCl in **(A)** wild-type and **(B)** *cop1-4* guard cells. **(C)** Average steady-state current-voltage relationships for wild-type and *cop1-4* guard cells. Current magnitudes were not statistically significantly different between *cop1-4* and wild-type (*P* = 0.346 at –180 mV). Student’s *t*-test was used. Error bars show ±SE.

**Figure 8:**
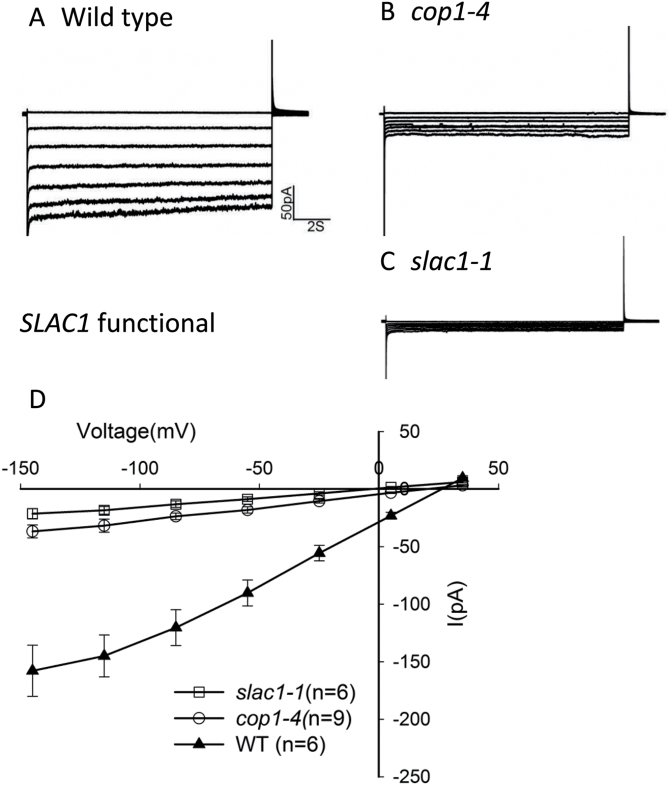
*cop1-4* Guard Cells Exhibit Reduced Ca^2+^-Activated S-Type Anion Channel Currents. Whole-cell recordings of Ca^2+^-activated S-type anion channel currents with 2 μM free Ca^2+^ in pipette solution in **(A)** wild-type, **(B)** *cop1-4*, and **(C)** *slac1-1* guard cells. **(D)** Average current-voltage curves of wild-type, *cop1-4*, and *slac1-1*. Current magnitudes were statistically significantly different between *cop1-4* and wild-type (*P* < 0.001 at –145 mV). Student’s *t*-test was used. Error bars show ±SE.

COP1 function is required for microtubule destabilization, which is required for stomatal closure (Figure 3B; Eisinger et al., 2012a), and lack of COP1 activity leads to cytoskeletal and structural changes in favor of open stomata (see above). Is cytoskeletal organization the only important cellular process modulated by COP1 activity or are central players such as ion channels also dependent on COP1? To explore COP1 function in stomatal control further, we tested *cop1-4* guard cells for defects in the activities of inward-rectifying K+ channels, which provide a pathway for K^+^ uptake during stomatal opening, and for Ca^2+^-activated S-type anion channels, which are activated to drive stomatal closure. Patch-clamp experiments showed that inward-rectifying channel activity was not significantly different between the *cop1-4* mutant and wild-type (Figure 7). However, Ca^2+^-activated S-type anion channel activity was dramatically down-regulated in *cop1-4* mutant guard cell (Figure 8). Thus, COP1 function is required for normal activation of Ca^2+^-activated S-type anion channels. These data are consistent with a critical role of COP1 in regulating stomatal closure.

## DISCUSSION

Stomata have been studied for at least 300 years (reviewed in Meidner, 1987). Multiple fields of study, including biochemistry, electrophysiology, and cell biology, have significantly advanced our knowledge of how stomata function, but the links between the mechanisms studied by these fields remain fragmented. Coordinated efforts between specialists in the different fields of study are needed in order to understand how stomatal pores respond to internal and external signals to control gas exchange and water loss through transpiration.

The roles of ion channels and related cellular signaling pathways in stomatal function have been well studied (Hetherington and Woodward, 2003; Kim et al., 2010), with signaling to the S-type anion channel SLAC1 being required for ABA-induced stomatal closure (Pei et al., 1997; Negi et al., 2008; Vahisalu et al., 2008). Our data indicate that stomatal closure also requires COP1 function and microtubule destabilization, and that both microtubule destabilization and S-type anion channel activity require COP1 function. Thus, COP1 may act upstream of S-type anion channels and microtubule destabilization to modulate stomatal closure. COP1 is well studied for its role in photomorphogenesis, where it acts during the night to destabilize factors involved in light signal transduction. At the onset of dawn, photoreceptors trigger COP1 exclusion from the nucleus, resulting in the stabilization of COP1 targets (Osterlund and Deng, 1998). Previously, it was reported that COP1 is required for stomatal closure in the dark (Mao et al., 2005). In our experiments, stomata of *cop1* mutants fail to close in response to ABA treatment as well (Figure 3A), indicating a role for COP1 in dark and ABA regulation of stomatal function. These results are consistent with a recent finding that COP1-targeted transcription factors HY5 and BBX21 are implicated in convergence of light and ABA signaling on the *ABI5* (*ABA INSENSITIVE 5*) promoter (Xu et al., 2014).

### COP1-Pathway Signaling Proteins May Be Involved in Stomatal Function

Our understanding of how COP1 regulates photomorphogenesis has advanced significantly in the past 20 years, but how COP1 mediates stomatal closure in the dark or in response to ABA remains to be defined. COP1 targets several proteins that promote light-induced processes for degradation through the 26S proteasome pathway (Yi and Deng, 2005; Li et al., 2011). While some of the COP1 targets are predominantly involved in signaling as modulated by red and far-red light, others like HY5 integrate signals from multiple photoreceptors, including phytochromes and crytochromes (Li et al., 2011). The SPA (SUPPRESSOR OF PHYTOCHROME A) proteins, which physically interact with COP1, are known mediators of COP1 activity and the *spa1 spa2 spa3 spa4* quadruple mutant displays *cop* phenotypes (see Hoecker, 2005). SPA1 interacts with COP1 to promote the degradation of specific targets, including HY5. Blue light-activated CRY1 interacts with SPA1 to release COP1 from the SPA1–COP1 complex and suppress COP1 activity (Liu et al., 2011). SPA1/SPA2 and SPA3/SPA4 form two subclasses within the SPA gene family (Laubinger and Hoecker, 2003). One or more of these known COP1 effectors and/or targets may be involved in stomatal closure in the dark, or there may be as-yet unknown effectors of COP1 specific to guard cell function. Light signaling and ABA signaling act in opposition to each other in regulating stomatal function. Thus, a further question is whether COP1 mediates ABA signaling to closure or if loss of COP1 and subsequent derepression of light-signaling pathways override ABA signaling.

COP1 may be involved in regulating the stability of transcriptional regulators in guard cells. The transcription factors MYB60 and MYB61 have been found to promote (Cominelli et al., 2005) and inhibit stomatal opening (Liang et al., 2005), respectively. They or other transcriptional regulators could be possible targets for direct COP1 action.

### COP1 Modulates S-Type Anion Channels

The *cop1* mutation had no significant effect on inward-rectifying K^+^ channel activity (Figure 7). Previously, it has been shown that transiently expressed KAT1:GFP forms a radially striped pattern in the plasma membrane of *Vicia faba* guard cells, and this pattern was found to be independent of the stomatal aperture and the cytoskeleton (Homann et al., 2007). Trafficking of inward-rectifying K^+^ channels in the plasma membrane through sequestration and recycling from endosomal membrane pool, and involvement of ABA and high turgor in regulating the K^+^ channel traffic has been well studied (Hurst et al., 2004; Meckel et al., 2004; Sutter et al., 2006, 2007; Eisenach et al., 2012). Stomatal closure is accompanied by internalization of K^+^ channels, and it remains to be seen whether COP1 is involved in stomatal re-opening possibly by influencing K^+^ channel recycling and/or activity.

In the absence of COP1 function, there was a significant reduction in S-type anion channel activity, which is largely encoded by the *SLAC1* gene (Figure 8). This might be accomplished either if COP1 promotes a positive regulator of S-type anion channel activity or removes a repressor of S-type anion channel activity. Candidates for such downstream targets are the OST1 or Ca^2+^-dependent protein kinases, which phosphorylate SLAC1 and are required for its activity, and the PP2C type protein phosphatases, including ABI1, which act to prevent SLAC1 activation (Geiger et al., 2009; Lee et al., 2009; Brandt et al., 2012). It is not yet known whether the abundance of SLAC1 is altered in *cop1* mutants. Links between COP1 and the activity of SLAC1 or other S-type anion channels remain to be drawn in the future.

### COP1-Mediates Cytoskeletal Organization in Guard Cells

Based upon our data, light acts to stabilize cortical microtubule arrays in guard cells and COP1 functions antagonistically. The mechanisms involved remain to be discovered. TUA6–GFP fluorescence is expected to be the same quantitatively, as tubulin polymer is degraded to tubulin monomer. Since GFP-tubulin signal is reduced in guard cells in darkness or after ABA treatment, relatively direct targeting of tubulin degradation itself is one possibility. A second possibility is down-regulation of factors that stabilize cortical microtubules. Possible downstream targets include the SPIRAL1 (SPR1) (Wang et al., 2011a) and WAVE-DAMPENED 2-LIKE3 (WDL3) (Liu et al., 2013) proteins—two microtubule-stabilizing proteins that appear to be degraded by the 26S proteasome pathway. SPR1 is a tip tracking protein unique to higher plants (Nakajima et al., 2004; Sedbrook et al., 2004), which is degraded upon salt stress (Wang et al., 2011b). WDL3 belongs to the WVD2/WDL family of microtubule regulatory proteins (Yuen et al., 2003; Perrin et al., 2007). WDL3 is degraded in the dark and its abundance in light was necessary for microtubule reorientation and inhibition of hypocotyl elongation (Liu et al., 2013).

Up-regulation of a destabilizing function is another possibility. An atypical mitogen-activated protein kinase-phosphatase, PHS1 (PROPYZAMIDEHYPERSENSITIVE1), phosphorylates Thr349 of α-tubulin at the inter-dimer interface to disrupt the polymerization capability of tubulin in *Arabidopsis* (Fujita et al., 2013). The kinase activity of PHS1 is suppressed by the intrinsic phosphatase activity, but the suppression of the kinase is removed by osmotic stress leading to near complete microtubule depolymerization (Fujita et al., 2013). The minimal peptide responsible for this activity has been identified, and acts as a potent and unregulated depolymerizing agent when overexpressed. COP1 may control factors involved in promoting PHS1 amount or tuning down the auto-inhibitory PHS1 phosphatase activity.

### Microtubule Organization Plays a Role in Guard Cells

The role of microtubule organization in both guard cell opening and closing remains an outstanding question. The observation that disassembly of cortical microtubules by the drug oryzalin causes stomatal closure in several conditions that otherwise stimulate opening or prevent closure, including *cop1* mutants, suggested that microtubule disassembly is generally critical for closure and may therefore be an important target of pathways signaling for closure. The cortical microtubule cytoskeletal has been shown in recent years to have at least three functions in the context of cell growth and morphogenesis: the positioning of cellulose synthase insertion into the plasma membrane (Gutierrez et al., 2009), the guidance of cellulose synthase as it deposits cellulose into the cell wall (Paradez et al., 2006), and the sequestering of trafficking compartments containing CESA during conditions of stress (Gutierrez et al., 2009). In the context of guard cell function, cortical microtubules could support the activities of essential proteins in the plasma membrane by regulating their trafficking, positioning, their ability to associate with signaling factors, or perhaps even as a direct regulator of their activity.

COP1 modulates both microtubule organization and S-type anion channel activity, which may require common or distinct downstream COP1-dependent pathways. It is clear that microtubule destabilization is required for stomatal closure. It is not yet known whether there are dependencies between S-type anion channels and microtubule array function. S-type anion channels and microtubules may function independently, or they may act together to regulate guard cell function.

### Guard Cell Shape

Stomatal apertures vary widely according to prevailing conditions, reflecting some sort of optimal balance between opening and closing functions. In the *cop1* mutant, the absence of any contribution by the closing mechanism might well account for the extreme opening of the stomata and their circular appearance. Further studies are required to understand how COP1 mediates guard cell shape.

### Intersection of Biochemical, Electrophysiological, and Cytoskeletal Pathways

COP1 appears to play a central role in regulating stomatal closure. Its influence on S-type anion channel activity, on microtubule organization, and guard cell shape reveals previously unknown functions for COP1. COP1 has been studied extensively as a critical destabilizer of photomorphogenesis-promoting factors. Data presented here suggest that COP1 acts in guard cells, playing an essential role through the plant life. Future studies are needed to resolve exactly how COP1 integrates signaling responses in guard cells by fine-tuning signaling mechanisms in favor of stomatal closure.

## METHODS

### Plant Material and Growth Conditions

*Arabidopsis thaliana* plants were grown in greenhouses under a minimum of 16-h photoperiod in natural light supplemented with artificial lights (75 μmol m^–2^ s^–1^). Temperature was maintained approximately to 22°C during the day and 20°C at night with heaters and evaporational cooling systems. Age-matched leaves (4–6 weeks old) were used for all of the experiments as previously described (Eisinger et al., 2012a, 2012b).

### Plant Lines Used

Col-0 *Arabidopsis* plants expressing *35S::GFP–TUA6* supplied by Takashi Hashimoto (Nara Institute of Science and Technology, Nara, Japan) were used to visualize tubulin. *35S::GFP–TUA6*×*cop1-4* crosses were performed, the F2 allowed to self, and F3 progeny scored for potential *35S::GFP–TUA6*,*cop1-4* homozygous individuals by phenotypic analysis for *cop1* morphogenetic phenotype and by expression of GFP–TUA6.

### Microscopy

Images were acquired with a Leica DM6000 inverted fluorescence microscope equipped with a Leica 63× n.a.=1.3 glycerin immersion objective lens and a Yokogawa CSU-10 spinning-disk confocal head (Gutierrez et al., 2009). The 488-nm line from an argon ion laser at 4 mW at the end of the fiber was used to excite GFP, and a band-pass filter (525/50, Semrock, Inc., IDEX Corporation, Illinois) was used as the emission filter. Images were captured with an Andor iXon3 EMCCD camera with an exposure time of 400 ms, intensification setting of 300. Z-stacks were acquired with a 0.3-μm step size. Slidebook software (Intelligent Imaging Innovations, Denver, CO) was used for image acquisition.

### Quantification of Fluorescence

GFP-Tubulin fluorescence was quantified from confocal image Z-stacks using ImageJ 1.48a (Wayne Rasband, NIH). Total fluorescence was quantified in sub-stacks (brightest point projections from consecutive planes) containing cortical microtubules in guard cells and pavement cells. An oval template was drawn around the pair of guard cells and the same template was used to quantify fluorescence in the neighboring pavement cells for consistency in area and volume measured for each cell type per sub-stack.

### Measurement of Stomatal Apertures

For each experiment, age-matched *Arabidopsis* leaves were excised from two or more individual plants with the same sowing dates grown in the greenhouse (as described above). All epidermal peel preparations and their treatments were performed in the morning. After each treatment as described, the epidermal peels were fixed immediately in ethanol, following the method described by Lloyd (1908). Epidermal peels were mounted onto slides in the fixative to image stomata with a light microscope using brightfield. Stomatal apertures and guard cell dimensions were measured using ImageJ 1.48a (Wayne Rasband, NIH), calibrated with a 0.01-mm standard image.

### Patch Clamp in Guard Cell Protoplasts

Plant growth and isolation of *Arabidopsis* guard cell protoplasts for patch clamping were performed as described in Laanemets et al. (2013). Seeds of *Arabidopsis* mutants *cop1-4* and *slac1-1* and the wild-type accession, Columbia, were grown in soil under long-day conditions (16-h light/8-h dark, 20°C–22°C). Two rosette leaves from 4–6-week-old plants were used to prepare *Arabidopsis* guard cell protoplasts. Guard cell protoplasts were isolated by enzymatic digestion for 15.5 h at 25°C on a circular shaker at 40 rpm. The enzymatic solution contained 1% Cellulase R-10 (Yakult, lot #091120–02), 0.5% Macerozyme R-10 (Yakult, lot #091112–01), 0.1 mM KCl, 0.1 mM CaCl_2_, 0.5% BSA, 0.1% kanamycin sulfate, and 10 mM ascorbic acid, 500 mM D-mannitol, (pH 5.6 with KOH). Guard cell protoplasts were washed twice with washing solution including 0.1 mM KCl, 0.1 mM CaCl_2_, and 500 mM D-sorbitol (pH 5.6 with KOH) by centrifugation for 10min at 200 *g*. Guard cell protoplast suspensions were stored in a 50-ml centrifugal tube on ice before use. The whole-cell mode was used for patch-clamp electrophysiology as described previously (Pei et al., 1997). To measure inward-rectifying potassium channel currents, the pipette solution contained 30 mM KCl, 70 mM K-Glu, 2 mM MgCl_2_, 2 mM CaCl_2_, 6.7 mM EGTA, 5 mM Mg-ATP, and 10 mM HEPES (Tris, pH 7.1), with an osmolarity of 500 mmol kg^–1^, and the bath solution contained 30 mM KCl, 1 mM CaCl_2_, 2 mM MgCl_2_, and 10 mM MES (Tris, pH 5.6) with an osmolarity of 485 mmol kg^–1^. Osmolarity was adjusted by D-sorbitol. Voltage pulses –180 mV to +20 mV were applied in +20-mV incremental steps.

Ca^2+^ activation of S-type anion channel currents was analyzed as described previously (Mori et al., 2006). The pipette solution was composed of 150 mM CsCl, 2 mM MgCl_2_, 5.86 mM CaCl_2_, 6.7 mM EGTA, 5 mM Mg-ATP, and 10 mM HEPES (Tris, pH 7.1). The bath solution contained 30 mM CsCl, 2 mM MgCl_2_, 1 mM CaCl_2_, and 10 mM MES (Tris, pH 5.6) (Schroeder and Keller, 1992). The osmolarities of the pipette solution and bath solution were also adjusted by D-sorbitol to 500 mmol kg^–1^ and 485 mmol kg^–1^ for S-type anion channel current recording, respectively. Before whole-cell recordings, guard cell protoplasts were pre-treated for 30 min in the same bath solution but with 40 mM CaCl_2_ added to prime guard cells for cytosolic Ca^2+^ activation of S-type anion channels (Allen et al., 2002). After treatment, the bath solution was changed from 40 mM CaCl_2_ to 1 mM CaCl_2_. S-type anion channel currents were recorded 7–10 min after achieving access to the whole-cell configuration. Membrane voltage was stepped in pulses of –30 mV decremental steps from +35 mV to –145 mV.

## SUPPLEMENTARY DATA

Supplementary Data are available at *Molecular Plant Online.*

## FUNDING

This work was supported in part by NSF Grant (0843617) to W.R.B. and NIH Grant (GM060396) to J.I.S. The authors are grateful for this support.

## Supplementary Material

Supplementary Data

## References

[CIT0001] Allen G.J. Murata Y. Chu S.P. Nafisi M. Schroeder J.I (2002) Hypersensitivity of abscisic acid-induced cytosolic calcium increases in the *Arabidopsis* farnesyltransferase mutant era1-2. Plant Cell. 14, 1649–1662 12119381 10.1105/tpc.010448 PMC150713

[CIT0002] Barbier-Brygoo H. De Angeli A. Filleur S. Frachisse J.M. Gambale F. Thomine S. Wege S (2011) Anion channels/transporters in plants: from molecular bases to regulatory networks. Annu. Rev. Plant Biol. 62, 25–51 21275645 10.1146/annurev-arplant-042110-103741

[CIT0003] Berry J.A. Beerling D.J. Franks P.J (2010) Stomata: key players in the earth system, past and present. Curr. Opin. Plant Biol. 13, 223–240 10.1016/j.pbi.2010.04.013 20552724

[CIT0004] Blatt M.R. Armstrong F (1993) K+ channels of stomatal guard cells: abscisic-acid-evoked control of the outward-rectifier mediated by cytoplasmic pH. Planta. 191, 330–341

[CIT0005] Brandt B. Brodsky D.E. Xue S. Negi J. Iba K. Kangasjarvi J. Ghassemian M. Stephan A.B. Hu H. Schroeder J.I (2012) Reconstitution of abscisic acid activation of SLAC1 anion channel by CPK6 and OST1 kinases and branched ABI1 PP2C phosphatase action. Proc. Natl Acad. Sci. U S A. 109, 10593–10598 22689970 10.1073/pnas.1116590109 PMC3387046

[CIT0006] Cominelli E. Galbiati M. Vavasseur A. Conbti L. Vuylsteke M. Leonhardt N. Dellaporta S.L. Tonelli C (2005) A guard-cell-specific MYB transcription factor regulates stomatal movements and plant drought tolerance. Curr. Biol. 15, 1196–1200 16005291 10.1016/j.cub.2005.05.048

[CIT0007] Eisenach C. Chen Z.H. Grefen C. Blatt M.R (2012) The trafficking protein SYP121 of *Arabidopsis* connects programmed stomatal closure and K(+) channel activity with vegetative growth. Plant J. 69, 241–251 21914010 10.1111/j.1365-313X.2011.04786.x

[CIT0008] Eisinger W. Ehrhardt D. Briggs W (2012a) Microtubules are essential for guard-cell function in Vicia and *Arabidopsis* . Mol. Plant. 5, 601–610 22402260 10.1093/mp/sss002

[CIT0009] Eisinger W.R. Kirik V. Lewis C. Ehrhardt D.W. Briggs W.R (2012b) Quantitative changes in microtubule distribution correlate with guard cell function in *Arabidopsis* . Mol. Plant. 5, 716–725 22492121 10.1093/mp/sss033

[CIT0010] Eun S.O. Lee Y (1997) Actin filaments of guard cells are reorganized in response to light and abscisic acid. Plant Physiol. 115, 1491–1498 9414559 10.1104/pp.115.4.1491 PMC158614

[CIT0011] Fujita S. Pytela J. Hotta T. Kato T. Hamada T. Akamatsu R. Ishida Y. Kutsuna N. Hasezawa S. Nomura Y. (2013) An atypical tubulin kinase mediates stress-induced microtubule depolymerization in *Arabidopsis* . Curr. Biol. CB 23, 1969–1978 24120637 10.1016/j.cub.2013.08.006

[CIT0012] Geiger D. Scherzer S. Mumm P. Stange A. Marten I. Bauer H. Ache P. Matschi S. Liese A. Al-Rasheid K.A. (2009) Activity of guard cell anion channel SLAC1 is controlled by drought-stress signaling kinase-phosphatase pair. Proc. Natl Acad. Sci. U S A. 106, 21425–21430 19955405 10.1073/pnas.0912021106 PMC2795561

[CIT0013] Gray J (2005) Guard cells: transcription factors regulate stomatal movements. Curr. Biol. 15, 593–595 10.1016/j.cub.2005.07.039 16085479

[CIT0014] Gutierrez R. Lindeboom J.J. Paredez A.R. Emons A.M. Ehrhardt D.W (2009) *Arabidopsis* cortical microtubules position cellulose synthase delivery to the plasma membrane and interact with cellulose synthase trafficking compartments. Nature Cell Biol. 11, 797–806 19525940 10.1038/ncb1886

[CIT0015] Hardtke C.S. Deng X.W (2000) The cell biology of the COP/DET/FUS proteins: regulating proteolysis in photomorphogenesis and beyond? Plant Physiol. 124, 1548–1557 11115873 10.1104/pp.124.4.1548 PMC1539311

[CIT0016] Hetherington A.M. Woodward F.I (2003) The role of stomata in sensing and driving environmental change. Nature. 424, 901–908 12931178 10.1038/nature01843

[CIT0017] Hoecker U (2005) Regulated proteolysis in light signaling. Curr. Opin. Plant Biol. 8, 469–476 16039154 10.1016/j.pbi.2005.07.002

[CIT0018] Holm M. Ma L.G. Qu L.J. Deng X.W (2002) Two interacting bZIP proteins are direct targets of COP1-mediated control of light-dependent gene expression in *Arabidopsis* . Genes Dev. 16, 1247–1259 12023303 10.1101/gad.969702 PMC186273

[CIT0019] Homann U. Meckel T. Hewing J. Hutt M.T. Hurst A.C (2007) Distinct fluorescent pattern of KAT1::GFP in the plasma membrane of *Vicia faba* guard cells. Eur. J. Cell Biol. 86, 489–500 17602785 10.1016/j.ejcb.2007.05.003

[CIT0020] Hosy E. Vavasseur A. Mouline K. Dreyer I. Gaymard F. Poree F. Boucherez J. Lebaudy A. Bouchez D. Very A.A. (2003) The *Arabidopsis* outward K+ channel GORK is involved in regulation of stomatal movements and plant transpiration. Proc. Natl Acad. Sci. U S A. 100, 5549–5554 12671068 10.1073/pnas.0733970100 PMC154382

[CIT0021] Hwang J.-U. Eun S.-O. Lee Y (2000) Structure and function of actin filaments in mature guard cells. In Actin: A Dynamic Framework for Multiple Plant Cell Functions Staiger C.J. Baluska F. Volkmann D. Barlow P.W, eds (Dordrecht, The Netherlands Kluwer Academic Publishers), pp. 427–436

[CIT0022] Hwang J.U. Lee Y (2001) Abscisic acid-induced actin reorganization in guard cells of dayflower is mediated by cytosolic calcium levels and by protein kinase and protein phosphatase activities. Plant Physiol. 125, 2120–2128 11299391 10.1104/pp.125.4.2120 PMC88867

[CIT0023] Hurst A.C. Meckel T. Tayefeh S. Thiel G. Homann U (2004) Trafficking of the plant potassium inward rectifier KAT1 in guard cell protoplasts of *Vicia faba* . Plant J. 37, 391–397 14731259 10.1046/j.1365-313x.2003.01972.x

[CIT0024] Jang I.C. Yang J.Y. Seo H.S. Chua N.H (2005) HFR1 is targeted by COP1 E3 ligase for post-translational proteolysis during phytochrome A signaling. Genes Dev. 19, 593–602 15741320 10.1101/gad.1247205 PMC551579

[CIT0025] Keller B.U. Hedrich R. Raschke K (1989) Voltage-dependent anion channels in the plasma membrane of guard cells. Nature. 341, 450–453 10.1002/j.1460-2075.1990.tb07608.x PMC552158 1701140

[CIT0026] Kim T.H. Böhmer M. Hu H. Nishimura N. Schroeder J.I (2010) Guard cell signal transduction network: advances in understanding abscisic acid, CO_2_ and Ca^2+^ signaling. Annu. Rev. Plant. Biol. 61, 561–591 20192751 10.1146/annurev-arplant-042809-112226 PMC3056615

[CIT0027] Kinoshita T. Doi M. Suetsugu N. Kagawa T. Wada M. Shimazaki K (2001) Phot1 and phot2 mediate blue light regulation of stomatal opening. Nature. 414, 656–660 11740564 10.1038/414656a

[CIT0028] Kollist H. Jossier M. Laanemets K. Thomine S (2011) Anion channels in plant cells. FEBS J. 278, 4277–4292 21955597 10.1111/j.1742-4658.2011.08370.x

[CIT0029] Kollist H. Nuhkat M. Roelfsema M.R.G (2014) Closing gaps: linking elements that control stomatal movement. New Phytol, 6 May, 10.1111/nph.12832 10.1111/nph.12832 24800691

[CIT0030] Laanemets K. Wang Y.F. Lindgren O. Wu J. Nishimura N. Lee S. Caddell D. Merilo E. Brosche M. Kilk K. (2013) Mutations in the SLAC1 anion channel slow stomatal opening and severely reduce K+ uptake channel activity via enhanced cytosolic [Ca2+] and increased Ca2+ sensitivity of K+ uptake channels. New Phytol. 197, 88–98 23126621 10.1111/nph.12008 PMC3508330

[CIT0031] Laubinger S. Hoecker U (2003) The SPA1-like proteins SPA3 and SPA4 repress photomorphogenesis in the light. Plant J. 35, 373–385 12887588 10.1046/j.1365-313x.2003.01813.x

[CIT0032] Lee S.C. Lan W. Buchanan B.B. Luan S (2009) A protein kinase-phosphatase pair interacts with an ion channel to regulate ABA signaling in plant guard cells. Proc. Natl Acad. Sci. U S A. 106, 21419–21424 19955427 10.1073/pnas.0910601106 PMC2795491

[CIT0033] Leonhardt N. Kwak J.M. Robert N. Waner D. Leonhardt G. Schroeder J.I (2004) Microarray expression analyses of *Arabidopsis* guard cells and isolation of a recessive abscisic acid hypersensitive protein phosphatase 2C mutant. Plant Cell. 16, 596–615 14973164 10.1105/tpc.019000 PMC385275

[CIT0034] Li J. Li G. Wang H. Wang Deng X (2011) Phytochrome signaling mechanisms. The Arabidopsis Book. Am. Soc. Plant Biol. 9, e0148 10.1199/tab.0148 PMC3268501 22303272

[CIT0035] Liang Y.K. Dubos C. Dodd I.C. Holroyd G.H. Hetherington A.M. Campbell M.M (2005) AtMYB61, an R2R3-MYB transcription factor controlling stomatal aperture in *Arabidopsis thaliana* . Curr. Biol. CB 15, 1201–1206 16005292 10.1016/j.cub.2005.06.041

[CIT0036] Lindeboom J.J. Nakamura M. Hibbel A. Shundyak K. Gutierrez R. Ketelaar T. Emons A.M.C. Mulder B.M. Kirik V. Ehrhardt D.W (2013) A mechanism for reorientation of cortical microtubule arrays driven by microtubule severing. Science., 7 November, 10.1126/science.1245533 10.1126/science.1245533 24200811

[CIT0037] Liu B. Zuo Z. Liu H. Liu X. Lin C (2011) *Arabidopsis* cryptochrome 1 interacts with SPA1 to suppress COP1 activity in response to blue light. Genes Dev. 25, 1029–1034 21511871 10.1101/gad.2025011 PMC3093118

[CIT0038] Liu L.J. Zhang Y.C. Li Q.H. Sang Y. Mao J. Lian H.L. Wang L. Yang H.Q (2008) COP1-mediated ubiquitination of CONSTANS is implicated in cryptochrome regulation of flowering in *Arabidopsis* . Plant Cell. 20, 292–306 18296627 10.1105/tpc.107.057281 PMC2276438

[CIT0039] Liu X. Qin T. Ma Q. Sun J. Liu Z. Yuan M. Mao T (2013) Light-regulated hypocotyl elongation involves proteasome-dependent degradation of the microtubule regulatory protein WDL3 in *Arabidopsis* . Plant Cell. 25, 1740–1755 23653471 10.1105/tpc.113.112789 PMC3694703

[CIT0040] Lloyd F.E (1908) The physiology of stomata. Carnegie Inst. Wash. Pub. No. 82. See Loftfield, J.V.G. (1921). The behavior of stomata. Carnegie Institution of Washington Pub. No. 314, pp. 1–104

[CIT0041] MacRobbie E.A. Kurup S (2007) Signalling mechanisms in the regulation of vacuolar ion release in guard cells. New Phytol. 175, 630–640 17688580 10.1111/j.1469-8137.2007.02131.x

[CIT0042] Mao J. Zhang Y.C. Sang Y. Li Q.H. Yang H.Q (2005) From the cover: a role for *Arabidopsis* cryptochromes and COP1 in the regulation of stomatal opening. Proc. Natl Acad. Sci. U S A. 102, 12270–12275 16093319 10.1073/pnas.0501011102 PMC1189306

[CIT0043] Meckel T. Hurst A.C. Thiel G. Homann U (2004) Endocytosis against high turgor: intact guard cells of *Vicia faba* constitutively endocytose fluorescently labelled plasma membrane and GFP-tagged K-channel KAT1. Plant J. 39, 182–193 15225284 10.1111/j.1365-313X.2004.02119.x

[CIT0044] Meidner H (1987) Three hundred years of research into stomata. In Stomatal Function Zeiger E. Farquhar G. Cowan I, eds (Stanford: Stanford University Press), pp. 7–27

[CIT0045] Miedema H. Assmann S.M (1996) A membrane-delimited effect of internal pH on the K+ outward rectifier of *Vicia faba* guard cells. J. Membrane Biol. 154, 227–237 8952952 10.1007/s002329900147

[CIT0046] Mori I.C. Murata Y. Yang Y. Munemasa S. Wang Y.F. Andreoli S. Tiriac H. Alonso J.M. Harper J.F. Ecker J.R (2006) CDPKs CPK6 and CPK3 function in ABA regulation of guard cell S-type anion-and Ca^2+^-permeable channels and stomatal closure. PLoS Biol. 4, e327 17032064 10.1371/journal.pbio.0040327 PMC1592316

[CIT0047] Mustilli A.C. Merlot S. Vavasseur A. Fenzi F. Giraudat J (2002) *Arabidopsis* OST1 protein kinase mediates the regulation of stomatal aperture by abscisic acid and acts upstream of reactive oxygen species production. Plant Cell. 14, 3089–3099 12468729 10.1105/tpc.007906 PMC151204

[CIT0048] Nakajima K. Furutani I. Tachimoto H. Matsubara H. Hashimoto T (2004) SPIRAL1 encodes a plant-specific microtubule-localized protein required for directional control of rapidly expanding *Arabidopsis* cells. Plant Cell. 16, 1178–1190 15084720 10.1105/tpc.017830 PMC423208

[CIT0049] Negi J. Matsuda O. Nagasawa T. Oba Y. Takahashi H. Kawai-Yamada M. Uchimiya H. Hashimoto M. Iba K (2008) CO_2_ regulator SLAC1 and its homologues are essential for anion homeostasis in plant cells. Nature. 452, 483–486 18305482 10.1038/nature06720

[CIT0050] Okamoto M. Peterson F.C. Defries A. Park S.Y. Endo A. Nambara E. Volkman B.F. Cutler S.R (2013) Activation of dimeric ABA receptors elicits guard cell closure, ABA-regulated gene expression, and drought tolerance. Proc. Natl Acad. Sci. U S A. 110, 12132–12137 23818638 10.1073/pnas.1305919110 PMC3718107

[CIT0051] Osterlund M.T. Deng X.W (1998) Multiple photoreceptors mediate the light-induced reduction of GUS–COP1 from *Arabidopsis* hypocotyl nuclei. Plant J. 16, 201–208 9839465 10.1046/j.1365-313x.1998.00290.x

[CIT0052] Osterlund M.T. Hardtke C.S. Wei N. Deng X.W (2000) Targeted destabilization of HY5 during light-regulated development of *Arabidopsis* . Nature. 405, 462–466 10839542 10.1038/35013076

[CIT0053] Paradez A. Wright A. Ehrhardt D.W (2006) Microtubule cortical array organization and plant cell morphogenesis. Curr. Opin. Plant Biol. 9, 571–578 17010658 10.1016/j.pbi.2006.09.005

[CIT0054] Pei Z.M. Kuchitsu K. Ward J.M. Schwarz M. Schroeder J.I (1997) Differential abscisic acid regulation of guard cell slow anion channels in *Arabidopsis* wild-type and *abi1* and *abi2* mutants. Plant Cell. 9, 409–423 9090884 10.1105/tpc.9.3.409 PMC156927

[CIT0055] Perrin R.M. Wang Y. Yuen C.Y. Will J. Masson P.H (2007) WVD2 is a novel microtubule-associated protein in *Arabidopsis thaliana* . Plant J. 49, 961–971 17319849 10.1111/j.1365-313X.2006.03015.x

[CIT0056] Saijo Y. Sullivan J.A. Wang H. Yang J. Shen Y. Rubio V. Ma L. Hoecker U. Deng X.W (2003) The COP1–SPA1 interaction defines a critical step in phytochrome A-mediated regulation of HY5 activity. Genes Dev. 17, 2642–2647 14597662 10.1101/gad.1122903 PMC280614

[CIT0057] Schroeder J.I. Hagiwara S (1989) Cytosolic calcium regulates ion channels in the plasma membrane of *Vicia faba* guard cells. Nature. 338, 427–430

[CIT0059] Schroeder J.I. Keller B.U (1992) Two types of anion channel currents in guard cells with distinct voltage regulation. Proc. Natl Acad. Sci. U S A. 89, 5025–5029 1375754 10.1073/pnas.89.11.5025 PMC49221

[CIT0060] Schroeder J.I. Raschke K. Neher E (1987) Voltage dependence of K channels in guard-cell protoplasts. Proc. Natl Acad. Sci. U S A. 84, 4108–4112 16593851 10.1073/pnas.84.12.4108 PMC305032

[CIT0061] Sedbrook J.C. Ehrhardt D.W. Fisher S.E. Scheible W.R. Somerville C.R (2004) The *Arabidopsis* sku6/spiral1 gene encodes a plus end-localized microtubule-interacting protein involved in directional cell expansion. Plant Cell. 16, 1506–1520 15155883 10.1105/tpc.020644 PMC490042

[CIT0062] Seo H.S. Yang J.Y. Ishikawa M. Bolle C. Ballesteros M.L. Chua N.H (2003) LAF1 ubiquitination by COP1 controls photomorphogenesis and is stimulated by SPA1. Nature. 423, 995–999 12827204 10.1038/nature01696

[CIT0063] Sutter J.U. Campanoni P. Tyrrell M. Blatt M.R (2006) Selective mobility and sensitivity to SNAREs is exhibited by the *Arabidopsis* KAT1 K+ channel at the plasma membrane. Plant Cell. 18, 935–954 16531497 10.1105/tpc.105.038950 PMC1425843

[CIT0064] Sutter J.U. Sieben C. Hartel A. Eisenach C. Thiel G. Blatt M.R (2007) Abscisic acid triggers the endocytosis of the *Arabidopsis* KAT1 K+ channel and its recycling to the plasma membrane. Curr. Biol. CB 17, 1396–1402 17683934 10.1016/j.cub.2007.07.020

[CIT0065] Vahisalu T. Kollist H. Wang Y.F. Nishimura N. Chan W.Y. Valerio G. Lamminmaki A. Brosche M. Moldau H. Desikan R. (2008) SLAC1 is required for plant guard cell S-type anion channel function in stomatal signalling. Nature. 452, 487–491 18305484 10.1038/nature06608 PMC2858982

[CIT0066] Wang R.S. Pandey S. Li S. Gookin T.E. Zhao Z. Albert R. Assmann S.M (2011a) Common and unique elements of the ABA-regulated transcriptome of *Arabidopsis* guard cells. BMC Genomics. 12, 216 21554708 10.1186/1471-2164-12-216 PMC3115880

[CIT0067] Wang S. Kurepa J. Hashimoto T. Smalle J.A (2011b) Salt stress-induced disassembly of *Arabidopsis* cortical microtubule arrays involves 26S proteasome-dependent degradation of SPIRAL1. Plant Cell. 23, 3412–3427 21954463 10.1105/tpc.111.089920 PMC3203425

[CIT0068] Wei N. Deng X.W (1996) The role of the COP/DET/FUS genes in light control of *Arabidopsis* seedling development. Plant Physiol. 112, 871–878 8938399 10.1104/pp.112.3.871 PMC158013

[CIT0069] Xu D. Li J. Gangappa S.N. Hettiarachchi C. Lin F. Andersson M.X. Jiang Y. Deng X.W. Holm M (2014) Convergence of Light and ABA signaling on the ABI5 promoter. PLoS Genetics. 10, e1004197 24586210 10.1371/journal.pgen.1004197 PMC3937224

[CIT0070] Yi C. Deng X.W (2005) COP1—from plant photomorphogenesis to mammalian tumorigenesis. Trends Cell Biol. 15, 618–625 16198569 10.1016/j.tcb.2005.09.007

[CIT0071] Yuen C.Y. Pearlman R.S. Silo-Suh L. Hilson P. Carroll K.L. Masson P.H (2003) WVD2 and WDL1 modulate helical organ growth and anisotropic cell expansion in *Arabidopsis* . Plant Physiol. 131, 493–506 12586874 10.1104/pp.015966 PMC166826

