# Supplementary material for: COP1 Jointly Modulates Cytoskeletal Processes and Electrophysiological Responses Required for Stomatal Closure
Source: Mol Plant. 2014 May 23;7(9):1441–54. doi: 10.1093/mp/ssu065 (PMC4153439; doi:10.1093/mp/ssu065)
Supplement: Supplementary Data [file supp_7_9_1441__index.html]

COP1 Jointly Modulates Cytoskeletal Processes and Electrophysiological Responses Required For Stomatal Closure — COP1 Jointly Modulates Cytoskeletal Processes and Electrophysiological Responses Required for Stomatal Closure — COP1 Jointly Modulates Cytoskeletal Processes and Electrophysiological Responses Required for Stomatal Closure — Supplementary Data 

# COP1 Jointly Modulates Cytoskeletal Processes and Electrophysiological Responses Required for Stomatal Closure

## Supplementary Data

Data files

**Files in this Data Supplement:**

- Supplementary Data - Supplementary Data
