# Supplementary material for: COP1 Jointly Modulates Cytoskeletal Processes and Electrophysiological Responses Required for Stomatal Closure
Source: Mol Plant. 2014 May 23;7(9):1441–54. doi: 10.1093/mp/ssu065 (PMC4153439; doi:10.1093/mp/ssu065)
Supplement: Supplementary Data [file supp_ssu065_Supplemental_Files_MPLANT_2014_025452.pdf]

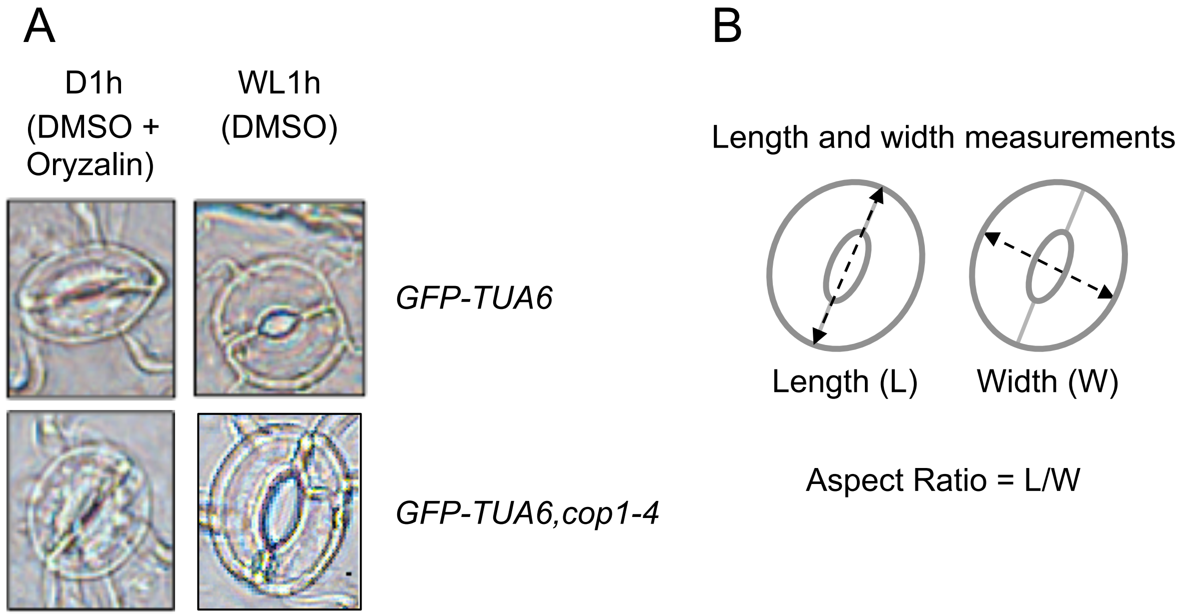

Supplementary Figure 1. Calculation of aspect ratio (Length/Width). (A) Representative images of closed and open *GFP-TUA6* and *GFP-TUA6,cop1-4* stomata. (B) Diagram showing stomatal length and width measurements that were used to calculate Aspect Ratios. Measurements were made using ImageJ, as described in Methods. Scale bar, 1 μm.

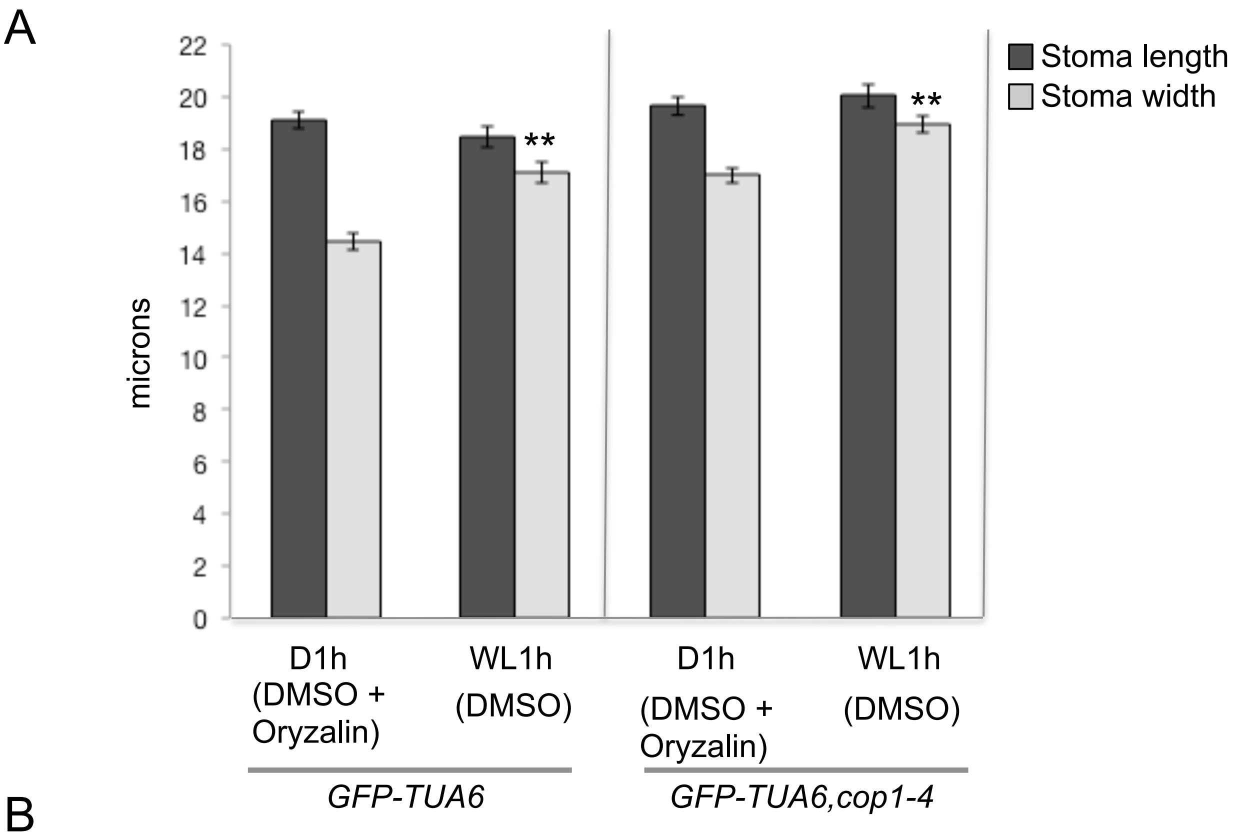

B

Aspect Ratio (Length/Width)	1.33	1.09	1.16	1.06
Change in Aspect Ratio	18.18%**		8.62%**	

Supplementary Figure 2. Dynamic range of stomatal movement (change in aspect ratio (L/W)) is reduced in *cop1-4* mutants. Length and width were measured of stomata in either closed (D1h in DMSO + oryzalin), or in open (WL1h in DMSO) positions. (A) Length and width of *GFP-TUA6* and *GFP-TUA6,cop1-4* stomata are plotted. (B) Aspect ratios and % changes in aspect ratio between closed and open stomata are shown. Data shown are from one of three independent experiments each with (n=20). Error bars show ±SE. Asterisks indicate ($P<0.001$, Student's *t* test) for the observed differences in width (A), and aspect ratio (B) between closed and open stomata determined for each genotype, showing that GFP-TUA6 stomata undergo a significantly larger change in shape than *GFP-TUA6,cop1-4* stomata.
